# Mechanism of Antibacterial Activity of Liposomal Linolenic Acid against *Helicobacter pylori*

**DOI:** 10.1371/journal.pone.0116519

**Published:** 2015-03-20

**Authors:** Sung Woo Jung, Soracha Thamphiwatana, Liangfang Zhang, Marygorret Obonyo

**Affiliations:** 1 Department of Medicine, University of California San Diego, La Jolla, United States of America; 2 Department of Internal Medicine, Korea University College of Medicine, Seoul, Korea; 3 Department of NanoEngineering, University of California San Diego, La Jolla, United States of America; Instituto de Tecnologia Quimica e Biologica, PORTUGAL

## Abstract

*Helicobacter pylori* infects approximately half of the world population and is a major cause of gastritis, peptic ulcer, and gastric cancer. Moreover, this bacterium has quickly developed resistance to all major antibiotics. Recently, we developed a novel liposomal linolenic acid (LipoLLA) formulation, which showed potent bactericidal activity against several clinical isolated antibiotic-resistant strains of *H*. *pylori* including both the spiral and coccoid form. In addition, LipoLLA had superior *in vivo* efficacy compared to the standard triple therapy. Our data showed that LipoLLA associated with *H*. *pylori* cell membrane. Therefore, in this study, we investigated the possible antibacterial mechanism of LipoLLA against *H*. *pylori*. The antibacterial activity of LipoLLA (C18:3) was compared to that of liposomal stearic acid (LipoSA, C18:0) and oleic acid (LipoOA, C18:1). LipoLLA showed the most potent bactericidal effect and completely killed *H*. *pylori* within 5 min. The permeability of the outer membrane of *H*. *pylori* increased when treated with LipoOA and LipoLLA. Moreover, by detecting released adenosine triphosphate (ATP) from bacteria, we found that bacterial plasma membrane of *H*. *pylori* treated with LipoLLA exhibited significantly higher permeability than those treated with LipoOA, resulting in bacteria cell death. Furthermore, LipoLLA caused structural changes in the bacterial membrane within 5 min affecting membrane integrity and leading to leakage of cytoplasmic contents, observed by both transmission electron microscopy (TEM) and scanning electron microscopy (SEM). Our findings showing rapid bactericidal effect of LipoLLA suggest it is a very promising new, effective anti-*H*. *pylori* agent.

## Introduction

*Helicobacter pylori* is known to be associated with the pathogenesis of gastric diseases [1]. Eradication of *H*. *pylori* is recommended for *H*. *pylori* associated diseases such as peptic ulcer disease, low grade gastric mucosa-associated lymphoid tissue lymphoma, and early gastric cancer, however the success rate of eradication with standard therapy has decreased with increasing antimicrobial resistance to *H*. *pylori* [2,3]. Although alternative treatments such as sequential or concomitant therapy using different combinations of known antibiotics have been extensively studied, they have shown conflicting efficacy results and antibiotic resistance remains an unsolved problem [4].

Free Fatty acids (FFAs) have both bactericidal and bacteriostatic activity against a broad range of bacteria [5,6,7]. In particular, several studies including ours have revealed that *H*. *pylori* is susceptible to FFAs including linolenic acid (LLA) *in vitro* [8,9,10,11,12,13,14]. However, the antibacterial effect of FFAs against *H*. *pylori in vivo* has remained unclear, since most of the effective FAs for *H*. *pylori* are poorly soluble and unstable. In addition, various FFA characteristics including carboxyl protonation, oxidation, esterification, and lipid–protein complexation in the gastric environment reduce the final concentration of FFAs at the mucus layer and therefore making them ineffective [6]. Indeed, a clinical study showed that orally ingested LLA did not inhibit the *H*. *pylori* colonization nor change the severity of *H*. *pylori* infection [15].

Recently, our laboratory used nanotechnology to solve the poor solubility of LLA in aqueous solution [12], This nano-platform liposome formulation was designed to directly release a high dose of LLA to the bacteria by fusion. The fusion of liposomal LLA (LipoLLA) with *H*. *pylori* was previously confirmed and showed comparable antibacterial efficacy against *H*. *pylori* with free LLA in inhibiting both spiral and coccoid forms of the bacteria *in vitro* [12]. In addition, we showed that LipoLLA was effective against several *H*. *pylori* clinical isolates including Shi470, SouthAfrica7, India7, Gambia94/24, PeCan4, Lithuania75, and SJM180 [12]. Further, we showed that LipoLLA penetrated the mucus layer of mouse stomach and was retained in the gastric lining. LipoLLA had superior *in vivo* antibacterial efficacy compared to the standard triple therapy in the mouse stomach [16].

**Fig 1:**
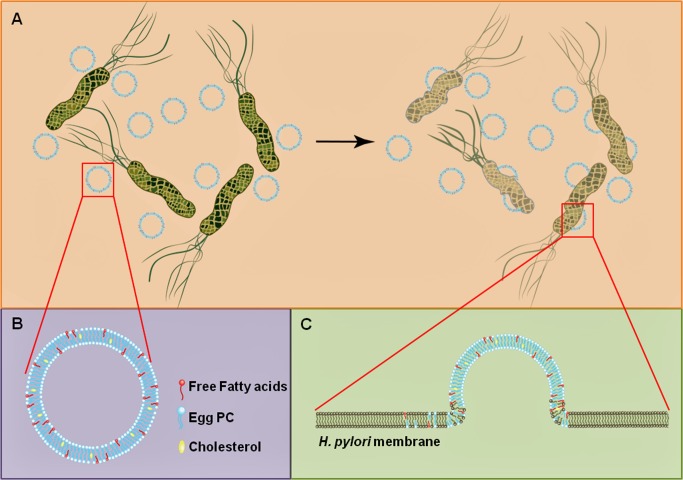
Schematic illustration of (A) *H*. *pylori* incubated with LipoFFA; (B) Structure and composition of LipoFFA consisting of phospholipid, cholesterol and FFA; (C) LipoFFA fuses with bacterial membrane for antibacterial activity.

Although mechanism of antibacterial activity of FFAs have not been fully elucidated yet, bacterial cell membrane has been regarded as the prime target [6]. Data from our previous study [12] suggested that LipoLLA killed *H*. *pylori* by targeting the cell membrane. The aim of this study therefore, was to determine the possible antibacterial mechanism of LipoLLA with a focus on the cell membrane of *H*. *pylori* (Fig. 1A). In addition, we compared the antibacterial efficacy against *H*. *pylori* of three C18 series of liposomal FFAs (LipoFFAs) including stearic acid (C18:0, SA), oleic acid (C18:1, OA), and linolenic acid (C18:3, LLA).

## Materials and Methods

### Materials

Hydrogenated L-α-phosphatidylcholine (Egg PC) and cholesterol were purchased from Avanti Polar Lipids, Inc. (Alabaster, AL). LLA was purchased from Ultra Scientific (North Kingstown, RI). Brain Heart Infusion (BHI), Columbia broth, and agar were purchased from Becton Dickinson (Sparks, MD). Horse blood was purchased from Hardy Diagnostics (Santa Maria, CA). BacTiter-Glo microbial cell viability assay kit was purchased from Promega Inc. (Madison, WI). SA, OA, 1-N-phenylnaphthylamine (NPN), and phosphate buffer saline (PBS) were obtained from SigmaAldrich Co. LLC (St. Louis, MO).

### Preparation and characterization of liposomal formulation of free fatty acids

Free fatty acid (FFA)-loaded liposomes were prepared by a well-characterized vesicle extrusion technique [12]. Briefly, liposomes composed of 10 mg of lipid components including Egg PC, cholesterol and FFAs such as SA, OA, or LLA (6:1:3, weight ratio) were dissolved in 3 mL of chloroform, which was then evaporated by leading nitrogen gas for 1 hour. The resultant dried lipid films were rehydrated with 10 mL of sterile PBS. The suspensions of lipid were vortexed for 3 min and then sonicated for 10 min in a bath sonicator (Fisher Scientific FS30D) to produce multilamellar vesicles (MLVs). A Ti-prove (Branson 450 sonifer) was used to sonicate the MLVs for 2 min at 20 W to produce small unilamellar vesicles (SUVs). The resulting SUVs were extruded through a 100 nm pore-sized polycarbonate membrane 11 times to form the final products of LipoSA, LipoOA, or LipoLLA. The size and zeta potential of LipoSA, LipoOA and LipoLLA were measured using the Malvern Zetasizer ZS (Malvern Instruments, UK). The mean diameter of LipoSA, LipoOA, and LipoLLA was determined through dynamic light scattering (DLS) and the zeta potential determined from electrophoretic mobility measurements [17]. Bare liposomes (liposomes without FFAs) composed of Egg PC and cholesterol (9:1, weight ratio) were used as a negative control. All characterization measurements were repeated three times at 25°C.

### H. pylori culture

A mouse-adapted Sydney strain 1 of *H*. *pylori*, originally described by Lee *et al*. [18] was used in this study. *H*. *pylori* was routinely grown on Columbia agar supplemented with 5% horse blood (Hardy Diagnostics LHB100) at 37°C under microaerobic conditions (10% CO_2_, 85% N_2_, and 5% O_2_) as previously described [19]. For liquid cultures for experiments, *H*. *pylori* obtained from agar plates were inoculated into Brain Heart Infusion (BHI) containing 5% fetal bovine serum (FBS) and incubated overnight at 37°C under microaerobic conditions with moderate reciprocal shaking.

### Bactericidal activity of liposomal fatty acids (LipoFFAs) against *H*. *pylori*

To determine the antimicrobial activity of LipoFFAs against *H*. *pylori*, a bacterial pellet was collected from an overnight liquid culture of *H*. *pylori* by centrifugation (3000 × g for 10 min) and resuspended in fresh BHI. The concentration of bacteria was measured by optical density at 600 nm (OD_600_), OD_600_ of 1.0 corresponding to approximately 1×10^8^ colony forming units (CFU)/mL. *H*. *pylori* (1×10^7^ CFU in 200 μL) were incubated with various concentrations of of LipoFFAs ranging from 62.5 to 1000 μg/mL in a 96-well plate at 37°C under microaerobic conditions on a reciprocal shaker for 30 min. For time-dependent antibacterial activity of LipoLLA, bacteria were incubated with 200, 300, or 400 μg/mL of LipoLLA for 5, 10, 20, or 30 min. After incubation, the samples were centrifuged (3000 × g) for 10 min and washed twice with PBS to remove residual LipoFFAs. The bacterial pellet was resuspended in PBS, followed by a series of 10-fold dilutions and the bacterial suspension inoculated onto Columbia agar plates supplemented with 5% laked horse blood. The agar plates were incubated for 4 days before counting colonies.

### Outer membrane permeability assay

The outer membrane permeabilization was assessed by measuring the uptake of NPN [20]. An overnight culture of *H*. *pylori* was harvested, washed and adjusted to an OD_600_ of 1.0 as described above. Approximately 5×10^6^ bacterial cells were treated with 400 μg/mL of LipoSA, LipoOA or LipoLLA for 5 min. Bare liposome and PBS were used as negative controls. After treatment, each bacterial suspension was centrifuged and resuspended in 200 μL of PBS. For NPN assay, 50 μL of NPN (20 mM final concentration) were added and mixed with treated bacteria. Fluorescence measurements were taken after shaking for 3 min, using a SPECTRAmax GEMINI EM microplate reader (Molecular Devices, Inc., Sunnyvale, CA) at an excitation wavelength of 350 nm and an emission wavelength of 420 nm. The NPN assays were performed at room temperature, repeated three times and the results expressed as relative fluorescence units [21].

### Plasma membrane permeability assay

Release of ATP from bacterial cells was measured by BacTiter-Glo microbial cell viability assay kit (Promega Inc.) as an indicator of plasma membrane permeability [22]. *H*. *pylori* were treated with 400 μg/mL of LipoSA, LipoOA, LipoLLA, or bare liposome for 5 min. One-milliliter from each sample containing approximately 1×10^6^ bacterial cells was centrifuged at 14,000 × g for 5 min and the supernatant containing released ATP from treated bacteria collected. BacTiter-Glo reagent (Promega Inc.) was added directly to the supernatants in each well of a 96-well plate at a ratio of 1:1. The mixture was shaken for 2 min and luminescence measured on a SPECTRAmax M2e microplate reader (Molecular Devices, Inc.).

### Transmission Electron Microscopy (TEM)

The effects of LipoLLA and LipoOA on the structure of *H*. *pylori were* examined with TEM. Briefly, an overnight culture of *H*. *pylori* was treated with LipoLLA or LipoOA (final concentration 400 μg/mL) and incubated for 5 or 30 min. In addition, *H*. *pylori* were treated with 200 μg/mL of LipoLLA for 30 min. PBS was used as negative control. Samples were centrifuged at 3000 × g for 5 min and bacterial pellets fixed by resuspending in 2% glutaraldehyde in 0.1 M sodium cacodylate buffer (pH 7.4). Bacterial pellets were then embedded in 2% agarose, post-fixed with 1% osmium tetroxide, and processed in Durcupan resin (Sigma-Aldrich). Fifty-five nm sections were examined using a FEI Tecnai G2 transmission electron microscope equipped with a 4K Eagle digital camera (FEI, Hilsboro, OR).

### Scanning Electron Microscopy (SEM)

Morphological changes of *H*. *pylori* treated with LipoLLA were observed by SEM. *H*. *pylori* was incubated with 400 μg/mL LipoLLA for 5 min and the bacteria harvested and processed for visualization by an FEIXL30 Environmental SEM. Bacteria were prepared for SEM as previously described [12]. Briefly, untreated and treated bacteria were centrifuged to remove the supernatant, and the remaining pellet was fixed with 2% glutaraldehyde for 2 hr at room temperature. Post fixing, the sample was centrifuged to remove glutaraldehyde and resuspended in 100 μL water. A bacterial suspension was spotted onto a polished silicon wafer and allowed to dry overnight in a biosafety cabinet. The samples were then coated with chromium before SEM imaging.

### Statistical analysis

Data from different groups were compared statistically using a Student’s t-test or two-way analysis of variance (ANOVA). Analyses were performed using GraphPad Prism 5.0 (GraphPad Software). *P* < 0.05 was considered significant.

## Results

### Characteristics and properties of LipoFFAs

**Fig 2:**
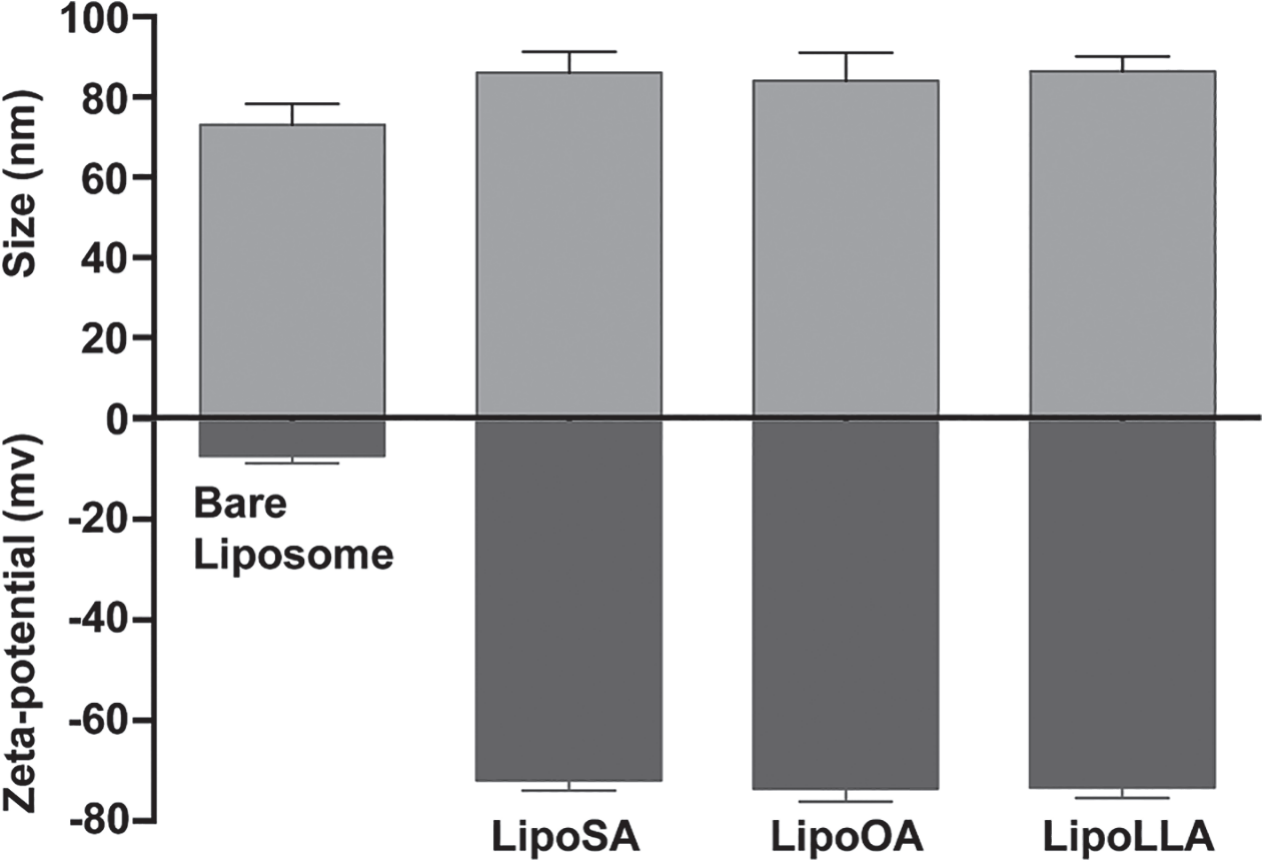
Comparison of hydrodynamic size and surface zeta potential of bare liposome, liposomal stearic acid (LipoSA), liposomal oleic acid (LipoOA) and liposomal linolenic acid acid (LipoLLA). All characterizations were performed using dynamic light scattering and each measurement was repeated three times at 25°C.

Liposomes made of egg PC, and cholesterol possess a lipid bilayer structure as illustrated in Fig. 1B. This bilayer structure of the liposome offers unique physiochemical properties for encapsulating and delivering FFAs such as SA, OA, and LLA. The amphiphilic FFAs molecules can be readily entrapped within the hydrophobic lipid bilayer of liposome by mixing FFAs with egg PC and cholesterol prior to liposome preparation. As a result, the LipoFFAs can overcome the poor solubility of FFAs in aqueous solution. For antibacterial applications, the liposome can fuse with bacterial membranes and thus directly release the entrapped FFAs molecules into bacterial membranes for efficient bactericidal activity as illustrated in Fig. 1C [12]. The loading yield of FFAs in liposome was determined by liquid chromatography–mass spectrometry following a procedure previously reported [23]. In this study, 30% by weight of FFAs in the initial mixture of egg PC, cholesterol, and FFAs resulted in a final encapsulation efficiency of approximately 20–30% [12,23]. There was a loss of FFAs during the experimental processes, including needle sonication, extrusion, and sterilization. The loss of FFAs during liposome preparation was also observed in our previous study to encapsulate LLA and OA into a liposomal formulation [23]. In the present study, LipoSA, LipoOA, LipoLLA, and bare liposome had a hydrodynamic size (diameter) of 86 ± 5, 84 ± 7, 86 ± 5, and 73 ± 5 nm, respectively. The changes in electrostatic potential profile across the membrane and surface, determined by electrophoretic mobility measurements, were used to confirm the incorporation of FFAs into the liposome membrane. As shown in Fig. 2, the surface ζ-potential of bare liposome was −7.3 ± 1.5 mV in deionized water. In contrast, the surface ζ-potential of LipoSA, LipoOA, and LipoLLA formulated were −72 ± 2, −74 ± 3, and −73 ± 2 mV, respectively. The decrease of surface ζ-potential was attributed to the incorporation of FFAs molecules, whose carboxylic acid group is deprotonated to COO^−^ at near pH of 7.4.

### Concentration-dependent bactericidal activity of LipoSA, LipoOA, and LipoLLA against *H*. *pylori*

**Fig 3:**
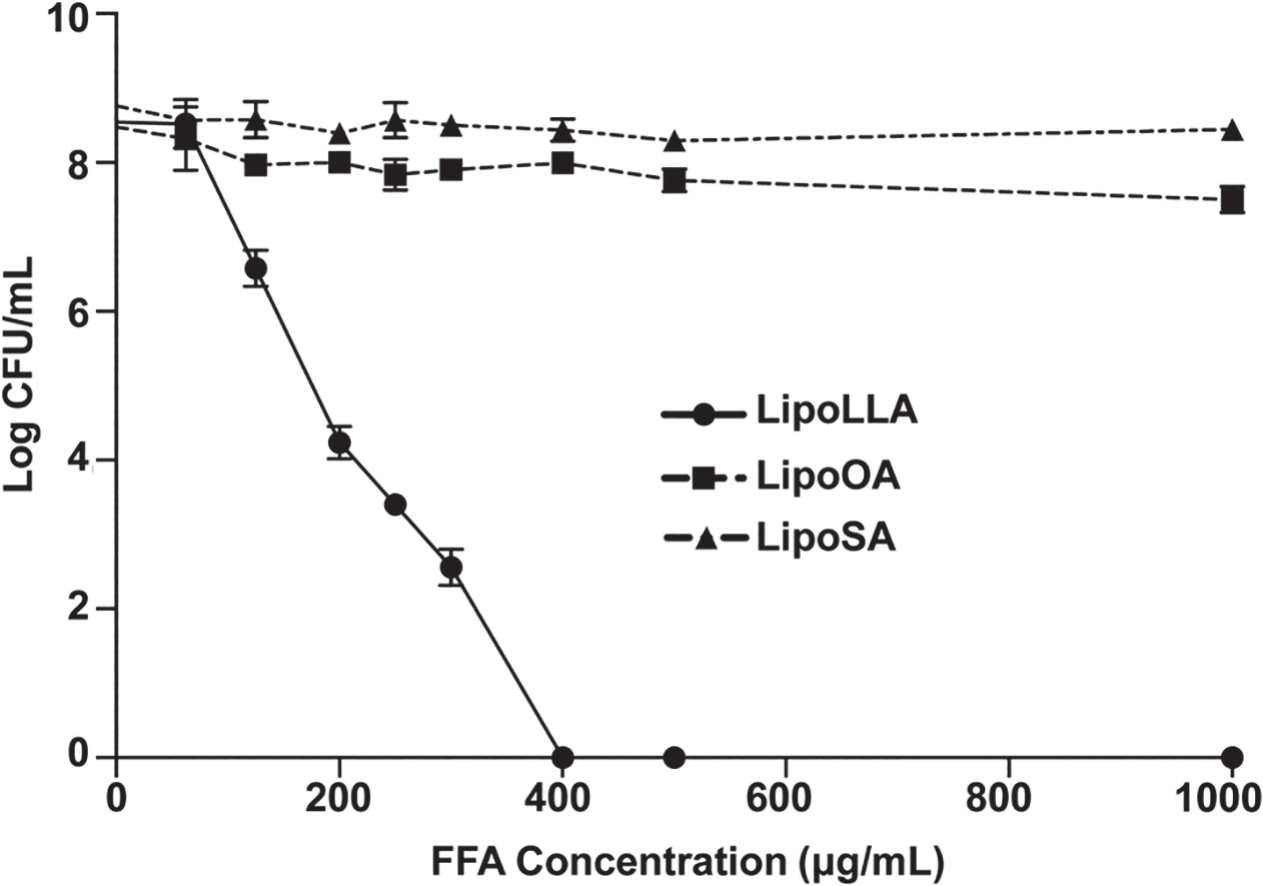
Concentration-dependent bactericidal activity of LipoLLA, LipoOA, and LipoSA against *H*. *pylori*. 5×10^6^ CFU/mL *H*. *pylori* was incubated with LipoLLA, LipoOA, or LipoSA at 37°C under microaerobic conditions for 30 min followed by inoculation onto Columbia agar plates for colony observation.

Antibacterial activities of LipoFFAs, including LipoLLA, LipoOA and LipoSA against *H*. *pylori* were evaluated *in vitro* by determining their minimal bactericidal concentrations (MBC) (Fig. 3). MBC for this study was defined as the minimal concentration that kills 99.99% (4 log) of targeted bacteria during 30 min incubation. Accordingly, the MBC value of LipoLLA was determined to be 200 μg/mL, where it killed 99.99% (~4 log) of *H*. *pylori*. When the LipoLLA concentration reached 400 μg/mL, no viable bacteria were detected. Meanwhile, 1 mg/mL of LipoOA killed 90% (~1 log) of *H*. *pylori* and LipoSA had no antibacterial effect against *H*. *pylori* at all concentrations tested up to 1 mg/mL.

### Time-dependent bactericidal activity of LipoLLA against *H*. *pylori*

**Fig 4:**
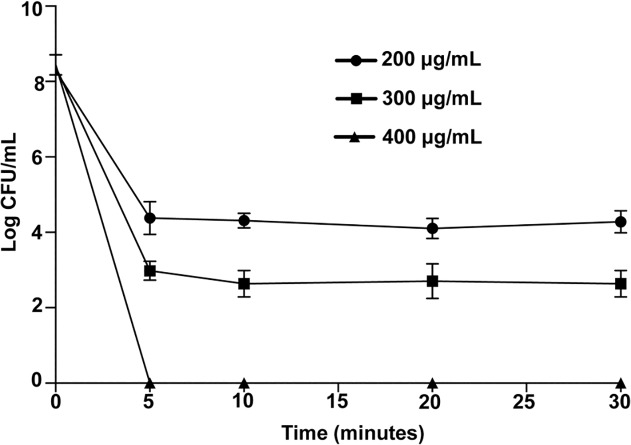
Time-dependent bactericidal activity of LipoLLA against *H*. *pylori*. 5×10^6^ CFU/mL *H*. *pylori* was incubated with 200, 300 or 400 g/mL LipoLLA at 37°C under microaerobic conditions for 5, 10, 20, or 30 min followed by inoculation onto Columbia agar plates for colony observation.

Due to the high potency exhibited by LipoLLA against *H*. *pylori*, we further investigated the time-kill kinetics of LipoLLA. A time-dependent antibacterial assay of LipoLLA was performed at three different concentrations (200, 300 or 400 g/mL) for 5, 10, 20, or 30 min. At all concentrations tested, LipoLLA rapidly decreased the number of viable bacteria within 5 min of incubation (Fig. 4). LipoLLA also exhibited concentration-dependent bactericidal effect within 5 min by killing 99.984% (3.8 log), and 999.999% (5.3 log) of *H*. *pylori* at 200, and 300 g/mL, respectively. No viable bacteria were detected, when *H*. *pylori* was treated with 400 g/mL LipoLLA for 5 min.

### Effect of LipoFFAs on the outer membrane of *H*. *pylori*

The antibacterial activity of LipoLLA occurred so rapidly that we hypothesized the antibacterial mechanism of LipoLLA was related to the bacterial membrane. NPN is a hydrophobic fluorescent probe whose quantum yield increases upon transfer from a hydrophilic to a hydrophobic environment. NPN is normally excluded by the outer membrane, but can enter into the hydrophobic interior of the outer membrane when membrane integrity is compromised [24]. Therefore, the NPN assay was used to determine the outer membrane permeability of *H*. *pylori* in response to lipoFFAs treatment. There was no difference in NPN uptake in LipoSA-treated *H*. *pylori* compared to the PBS control or bare liposome. *H*. *pylori* treated with LipoOA or LipoLLA had a significant (*P* < 0.001) increase in the fluorescence signal of NPN compared to PBS control, indicating that LipoOA and LipoLLA increased the outer membrane permeability of *H*. *pylori* (Table 1).

**Table 1:** Uptake of N-phenylnapthylamine (NPN) by *H*. *pylori* in the presence of bare liposome, LipoSA, LipoOA, or LipoLLA.

Sample	RFU (mean ±SD)
*H*. *pylori* + PBS	179.7 ± 19.9
*H*. *pylori* + bare liposome	236.6 ± 3.2
*H*. *pylori* + LipoSA	190.0 ± 3.3
*H*. *pylori* + LipoOA	1237.1 ± 58.3^a^
*H*. *pylori* + LipoLLA	845.1 ± 38.5 ^a^ ^,^ ^b^

^a^ Significantly different from controls (*P* < 0.001).

^b^ Significantly different from cells treated with LipoOA (*P* = 0.02).

RFU, relative fluorescence unit; SD, standard deviation

### Effect of LipoFFAs on the plasma membrane of *H*. *pylori*

**Fig 5:**
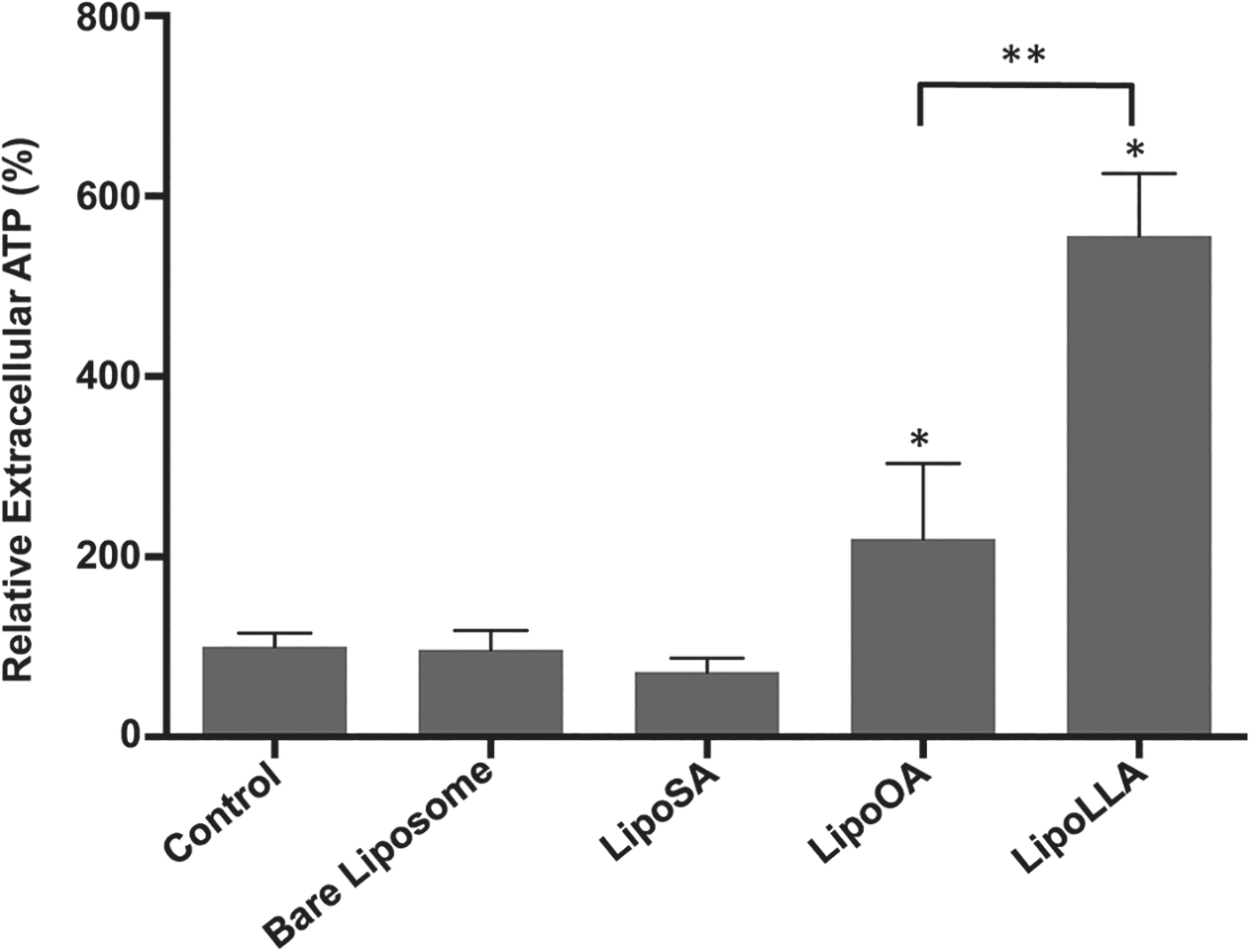
Release of ATP from *H*. *pylori* by liposomal fatty acids. *H*. *pylori* were treated for 5 min with the indicated compounds, and bacterial cells and supernatants separated by centrifugation. Supernatants were analyzed for their ATP content as described in Methods. *Significantly different from control (*P* < 0.01); **Significantly different between bacterial cells exposed to LipoOA and LipoLLA (*P* < 0.01).

Both LipoOA and LipoLLA increased outer membrane permeability of *H*. *pylori*, however, LipoLLA was significantly more effective in killing *H*. *pylori* than LipoOA. We therefore examined the effect of these LipoFFAs on the plasma membrane of *H*. *pylori*. A disrupted plasma membrane has been shown to result in immediate collapse of the proton gradient accompanied with a rapid release of intracellular components such as ATP into the medium [22]. We measured the release of ATP from the cytoplasm through the plasma membrane of lipoFFAAs-treated bacteria as an indicator of plasma membrane disruption. *H*. *pylori* treated with LipoSA released ATP at the same level as bacterial cell incubated with bare liposomes or control PBS (Fig. 5). Treatment with LipoLLA or LipoOA both significantly (*P*<0.01) increased ATP release from *H*. *pylori* compare to control. When the effects of the two LipoFFAs (LipoLLA and LipoOA) were compared directly, LipoLLA treatment resulted in significantly (*P* < 0.01) greater release of ATP than LipoOA (Fig. 5).

### LipoFFAs-induced morphological changes

**Fig 6:**
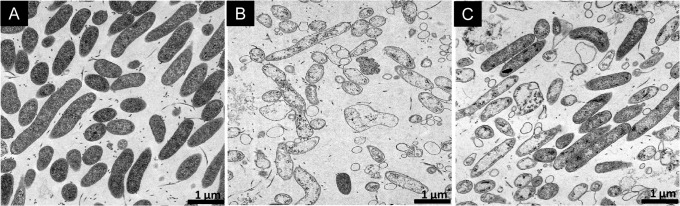
TEM images of *H*. *pylori* bacteria exposed to different treatments. (A) PBS, (B) LipoLLA and (C) LipoOA. In all experiments, the initial concentration of bacteria was 5 × 10^7^ CFU/mL, and the drug concentration used was 400 μg/mL. All samples were treated for 30 min before fixation.

**Fig 7:**
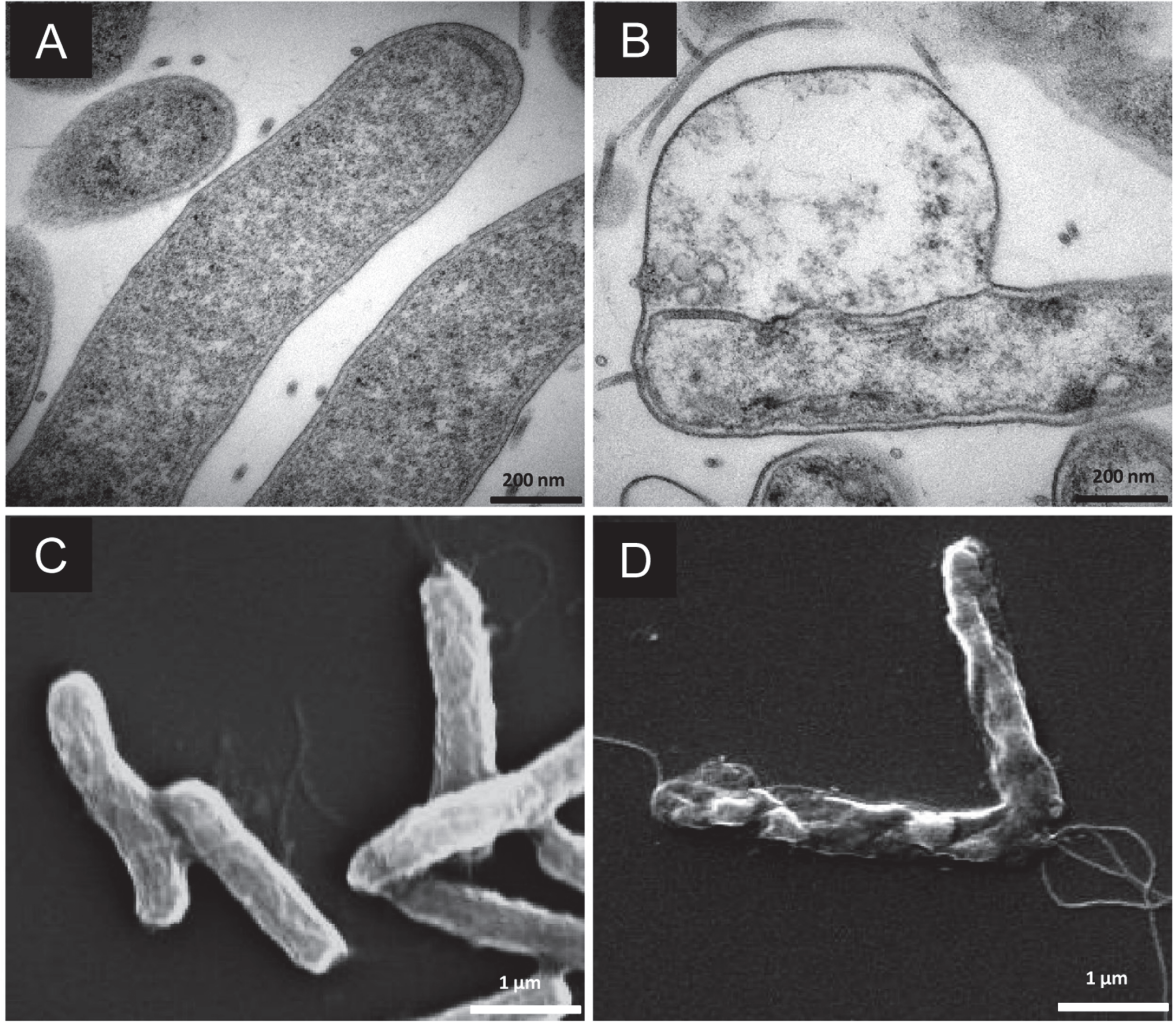
TEM (A and B) and SEM (C and D) images of *H*. *pylori* bacteria exposed to LipoLLA. Bacteria were treated for 5 min with PBS control (A and C) or LipoLLA (B and D). The concentration of bacteria used was 5 × 10^6^ CFU/mL and the drug concentration was 400 μg/mL.

Morphological changes of *H*. *pylori* exposed to LipoLLA or LipoOA for 30 min were observed by TEM. Untreated *H*. *pylori* were used as control. Untreated bacteria appeared normal with intact cell wall and dense cytoplasm (Fig. 6A). Bacteria exposed to LipoLLA showed loss of cytoplasmic contents and separation of the plasma membrane from the outer membrane, which formed blebs (Fig. 6B, S1 Fig. and S2 Fig.). Structural changes in bacteria exposed to LipoOA were less striking. Intact bacteria with dense cytoplasm were present concurrently with affected bacteria, which had similar morphologic changes to those caused by the LipoLLA (Fig. 6C). We also examined morphological changes of *H*. *pylori* when treated with LipoLLA for 5 min by SEM and compared the images with TEM images at higher magnification. The outer membrane of the untreated *H*. *pylori* adhered closely to the plasma membrane and enclosed a relatively homogeneous cytoplasm (Fig. 7A). Their cell surface appeared smooth and homogenous when examined under SEM (Fig. 7C and S3 Fig.). For LipoLLA-treated *H*. *pylori*, TEM images showed membrane detachments between the outer and plasma membrane accompanied with leakage of cytoplasmic contents into the intermembrane space (Fig. 7B). Concomitantly, SEM images displayed destructive outline caused by blistering of the outer membrane (Fig. 7D).

## Discussion

It is known that unsaturated FFAs tend to have greater potency in inhibiting the growth of bacteria than saturated FFAs with same length carbon chain [6]. Studies on antibacterial effect of saturated and unsaturated FFAs against *H*. *pylori* [8,9,10] suggest that bactericidal activity increases with degree of unsaturation [11]. In the present study, due to poor solubility in aqueous solution of FFAs, we incorporated FFAs, including SA, OA, and LLA, into liposomes. The size of liposomes is an important factor that determines their bactericidal mechanism [23,25]. Liposomes in the 50–100 nm size range have been shown to be relatively stable compared to the smaller liposomes (diameter, < 50 nm) while at the same time preserving high surface tension required to fuse with bacterial membrane [23]. When the liposomes fuse with bacterial membranes, the encapsulated FFAs are released into bacterial membranes with higher local antibacterial concentration, resulting in better efficient bactericidal activity. Treatment of *H*. *pylori* with LipoLLA resulted in a sharp decrease in viable bacterial numbers and this bactericidal activity was concentration dependent. In contrast, bactericidal activity of LipoOA or LipoSA against *H*. *pylori* was not influenced by their concentration. The bactericidal activity of LipoOA against *H*. *pylori* was much lower compared to that of LipoLLA while LipoSA had no effect against *H*. *pylori*. Moreover, LipoLLA rapidly killed *H*. *pylori* in 5 min. Our present results using liposomal formulation of FFAs were consistent with those of previous studies using FFAs, which showed a superior antibacterial effect of LLA when compared to OA [9]. However, in contrast to our study in which only 5min was sufficient for LipoLLA to completely kill *H*. *pylori*, incubation of FFAs with *H*. *pylori* required anywhere from 40 min to 48 hrs to achieve significant antibacterial effects [8,9,10,11,13,14] The high potency of LLA in our study can be explained by a unique capability of LipoLLA to fuse rapidly with the bacterial membrane followed by release of LLA all at once, resulting in membrane integrity disruption and causing bacterial death.

Fusion between bacterial membrane and LipoFFAs delivers FFA directly into the membrane, resulting in alteration of the phospholipid composition of the bacteria [11]. Incorporation of FFAs into the cell wall, therefore most probably affects permeability of the outer membrane. The NPN uptake assay is a standard method used to measure alterations in the permeability of the outer membrane of Gram-negative bacteria including *H*. *pylori* [21,26,27]. LipoLLA and LipoOA increased NPN fluorescence signal compared to control, revealing evidence of damage to the outer membrane. Interestingly, treatment of *H*. *pylori* with LipoOA resulted in higher NPN signal than LipoLLA contrary to expectations. This could be explained by the fact that LipoLLA rapidly killed *H*. *pylori* resulting in reduced viable bacteria during the determination of outer membrane permeability by the NPN uptake assay. Low numbers of bacteria would therefore lead to the reduced NPN uptake of LipoLLA treated bacteria, thereby resulting in a lower NPN signal than that of LipoOA treated bacteria. While LipoOA increased outer membrane permeability as evidenced by NPN uptake, it was not very effective in killing *H*. *pylori*. Permeabilization of the outer membrane is necessary but not sufficient to kill bacteria [28]. We therefore determined the effect of LipoFFAs on the plasma membrane permeability by measuring the release of ATP from treated bacteria. LipoLLA-treated bacterial cells released more intracellular ATP into the medium compared to LipoOA-treated bacteria. As expected there was no difference in ATP release between LipoSA-treated bacteria and controls. These results were consistent with data from antibacterial assay, which showed that LipoLLA was the most potent LipoFFAs against *H*. *pylori* followed by LipoOA while LipoSA had no bactericidal effects.

TEM images showing visual morphological changes of bacteria treated with LipoOA or LipoLLA were similar but the number of affected cells was less in LipoOA-treated samples. In addition, both TEM and SEM images from treated *H*. *pylori* exhibited membrane detachments after exposure to LipoLLA or LipoOA with worse effects observed in LipoLLA treated bacteria, which was consistent with data from the killing assay.

In the present study, LipoOA increased the outer membrane permeability of *H*. *pylori* but had weak effects on the permeability of the plasma membrane and hence poor killing of *H*. *pylori*. Potency of FFAs may be influenced by the FA present in lipopolysaccharides (LPS) and phospholipids of the membrane. While unsaturated FAs were not detected in the LPS of the outer membrane, some of them including OA were present in small amount in phospholipids of *H*. *pylori* [29], which may affect the antibacterial activity of OA. Indeed SA, which is known as one of the major fatty acids in LPS of *H*. *pylori* [29], had no effect on membrane permeability or growth of *H*. *pylori*.

In conclusion, LipoLLA is a potent bactericidal agent against *H*. *pylori* that permeabilizes and disrupts membranes and acts very rapidly in a dose-dependent manner. Due to this highly destructive mechanism of bacterial killing combined with our successful in vivo delivery system, LipoLLA could be a potential therapeutic agent against *H*. *pylori* infection.

## Supporting Information

S1 Fig TEM images of *H*. *pylori* bacteria exposed to LipoLLA (A and B). In all experiments, the initial concentration of bacteria was 5 × 10^7^ CFU/mL, and the drug concentration used was 200 μg/mL. All samples were treated for 30 min before fixation.
(TIF)

S2 Fig TEM images of *H*. *pylori* bacteria exposed to LipoLLA (A, B, C, D). In all experiments, the initial concentration of bacteria was 5 × 10^7^ CFU/mL, and the drug concentration used was 400 μg/mL. All samples were treated for 30 min before fixation.
(TIF)

S3 Fig SEM images of *H*. *pylori* bacteria exposed to LipoLLA (A, B, and C). Bacteria were treated for 5 min with LipoLLA. The concentration of bacteria used was 5 × 10^6^ CFU/mL and the drug concentration was 400 μg/mL.
(TIF)
