# Supplementary figures and images for: Mechanism of Antibacterial Activity of Liposomal Linolenic Acid against Helicobacter pylori
Source: PLoS One. 2015 Mar 20;10(3):e0116519. doi: 10.1371/journal.pone.0116519 (PMC4368202; doi:10.1371/journal.pone.0116519)

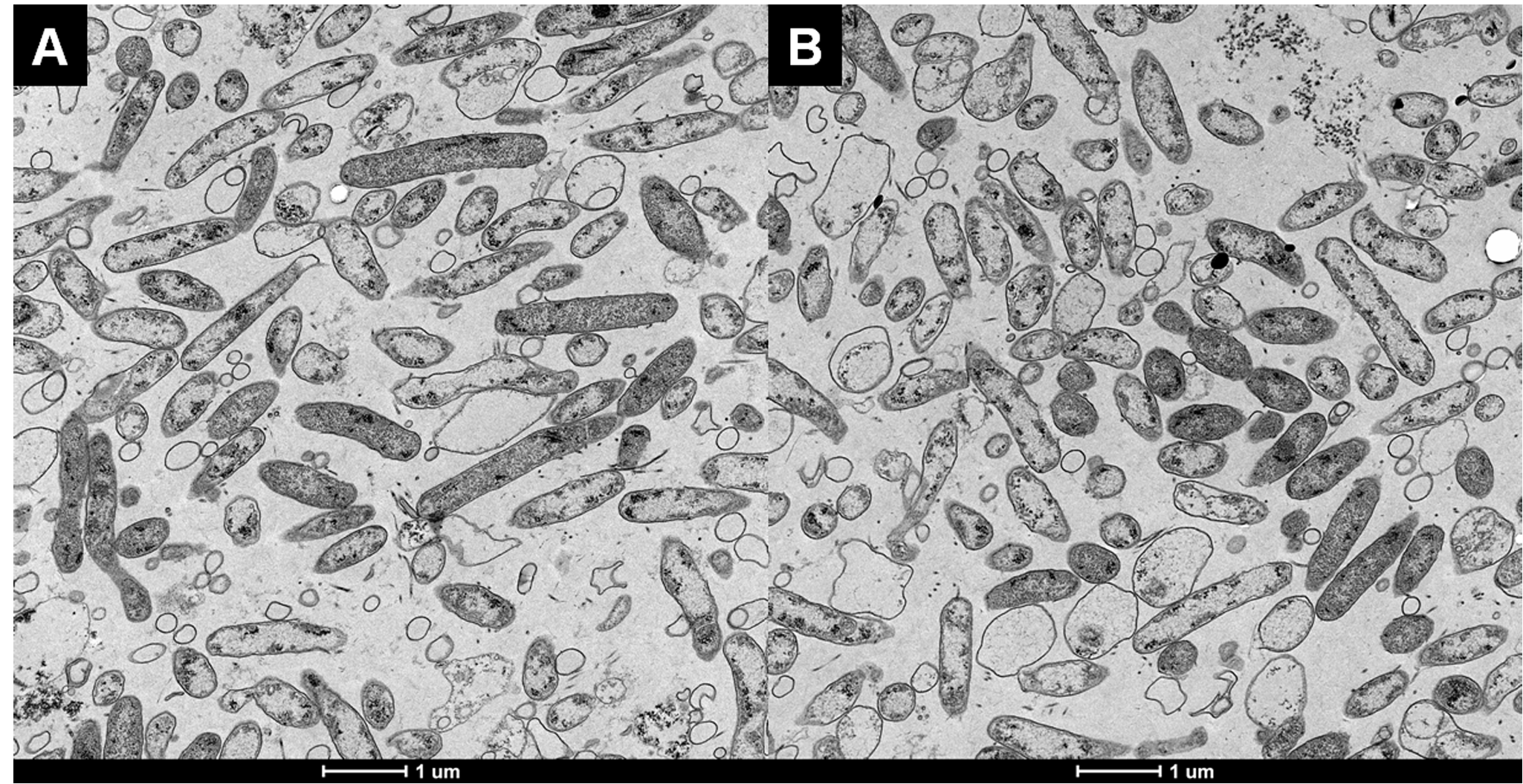

Supplement: S1 Fig — In all experiments, the initial concentration of bacteria was 5 × 10^7^ CFU/mL, and the drug concentration used was 200 μg/mL. All samples were treated for 30 min before fixation. (TIF) [file pone.0116519.s001.tif]

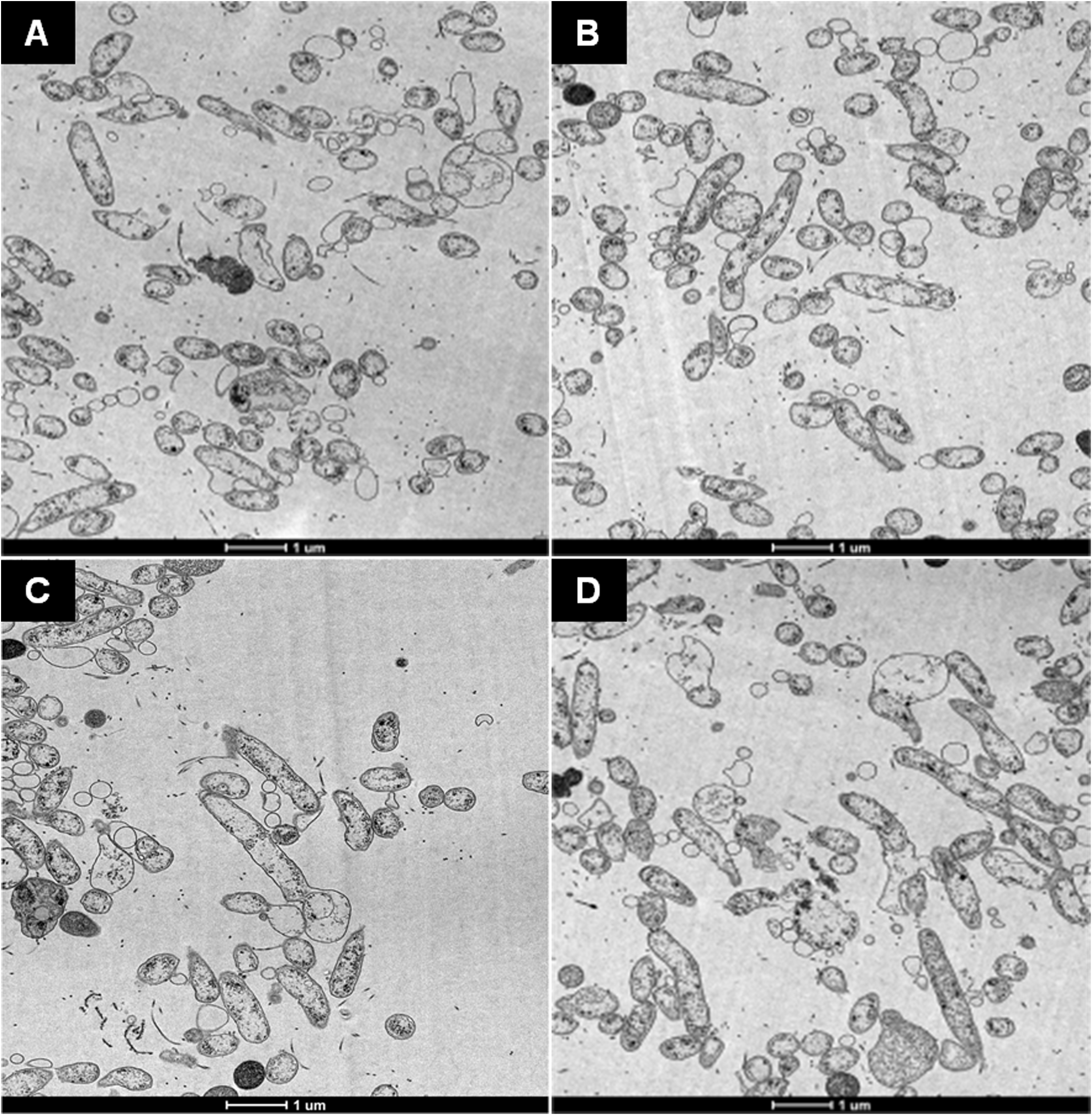

Supplement: S2 Fig — In all experiments, the initial concentration of bacteria was 5 × 10^7^ CFU/mL, and the drug concentration used was 400 μg/mL. All samples were treated for 30 min before fixation. (TIF) [file pone.0116519.s002.tif]

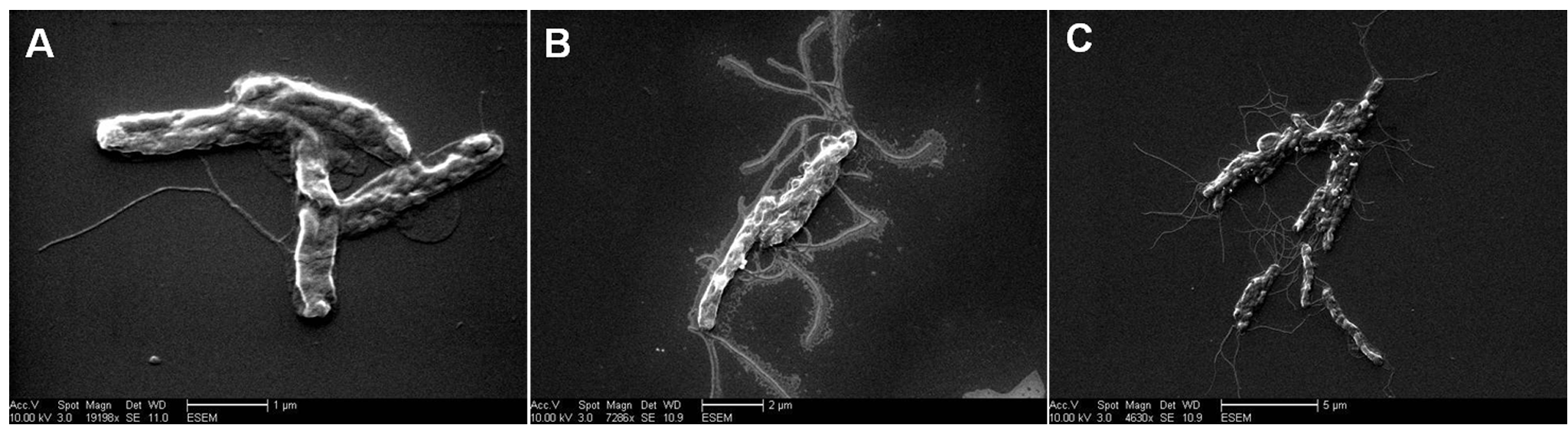

Supplement: S3 Fig — Bacteria were treated for 5 min with LipoLLA. The concentration of bacteria used was 5 × 10^6^ CFU/mL and the drug concentration was 400 μg/mL. (TIF) [file pone.0116519.s003.tif]
